# A Sarcoidosis Patient Presents with Adrenal Insufficiency: A Standardized Patient Scenario for Medical Students and Residents

**DOI:** 10.7759/cureus.2833

**Published:** 2018-06-18

**Authors:** Shahdi K Malakooti, Leslie V Simon

**Affiliations:** 1 Internal Medicine, Orange Park Medical Center, Orange Park, USA; 2 Emergency Medicine/ Medical Simulation, Mayo Clinic, Jacksonville, USA

**Keywords:** standardized patient scenario, adrenal crisis, adrenal insufficiency, case-based learning, sarcoidosis, corticosteroids

## Abstract

Introduction

The widespread use of corticosteroids for treatment of inflammatory conditions has resulted in the need to promptly recognize drug-induced adrenal insufficiency. This scenario was inspired by an actual case and aims to enhance critical thinking. Our case is unique as we use a case-based format with written tests to track progress.

Methods

A pre-assessment was conducted to measure baseline knowledge with residents and medical students. A standardized patient played a 70-year-old female with sarcoidosis who was in the emergency department with weakness and fatigue. The learners obtained her history whereby they discovered that she had recently stopped taking prednisone. They identified adrenal insufficiency and reinstated glucocorticoid therapy. The scenario lasted 10 minutes after which there were a debriefing session and post-debriefing assessment. All were completed in under one hour.

Results

Our pre-scenario assessment revealed that all learners had less knowledge of adrenal insufficiency than thyroid disease with average scores of 66.63% and 91.25%, respectively. The average score of the adrenal insufficiency test increased from 66.63% to 87.45% on the post-debriefing assessment and the largest improvement was seen in first-year residents. Assessments measured via the Likert scale determined that all learners found the case well-devised to contribute to their understanding of adrenal insufficiency.

Discussion

The largest improvement unexpectedly was seen in first-year residents which may be due to variations in repetition and retention of medical knowledge in the months prior to starting residency. This module is best suited for first-year internal medicine, family medicine, and emergency medicine residents and upper-level medical students.

## Introduction

Adrenal insufficiency is an endocrine disorder in which one or more of the hormones that are normally secreted by the adrenal gland (i.e., aldosterone, cortisol, dehydroepiandrosterone, epinephrine, norepinephrine) are not produced in adequate amounts [1]. Adrenal insufficiency can be categorized as primary, secondary, and tertiary depending on the origin of the disease [1].

The hypothalamus secretes corticotropin-releasing hormone (CRH) which stimulates the anterior pituitary to release adrenocorticotropic hormone (ACTH). ACTH then prompts the adrenal cortex to secrete cortisol. Cortisol itself self-regulates the axis via negative feedback to the hypothalamus and anterior pituitary to maintain cortisol levels in certain ranges throughout the day and in times of stress [2].

Primary adrenal insufficiency occurs due to adrenal gland dysfunction while secondary adrenal insufficiency is a result of anterior pituitary dysfunction [2]. Tertiary adrenal insufficiency, on the other hand, occurs due to hypothalamic dysfunction. Secondary and tertiary adrenal insufficiency are commonly drug-induced, whereby exogenous corticosteroids suppress the normal physiologic function of the hypothalamus and anterior pituitary in regulating cortisol levels [2]. This is an important physiologic process to understand because of the widespread use of steroids for a variety of autoimmune and inflammatory diseases.

Prednisone is an example of a corticosteroid commonly prescribed for its immunosuppressant properties and acts analogously to cortisol to suppress the hypothalamic-pituitary-adrenal axis [1]. During times of physiologic stress, the body responds with inflammatory cytokines and molecules that stimulate secretion of cortisol via numerous complex pathways [3]. Cortisol, in turn, reflexively stimulates gluconeogenesis to ensure adequate energy supplies, regulates ion transport, and works to maintain physiologic equilibrium [4]. If CRH and ACTH are suppressed, then discontinuation of prednisone may result in adrenal insufficiency and in times of physiologic stress may precipitate a life-threatening adrenal crisis (acute adrenal insufficiency) [5].

Drug-induced adrenal insufficiency can be difficult to diagnose since symptoms mimic other conditions and patients may fail to mention which medications they have been taking unless a full history is elicited by the clinician. In drug-induced acute adrenal insufficiency, the constellation of symptoms includes confusion, fatigue, weakness, nausea, abdominal pain, and dizziness from postural hypotension. These findings, as well as laboratory values showing hyponatremia, hypoglycemia, and pre-renal failure, should prompt the clinician to consider secondary or drug-induced adrenal crisis [5]. Primary adrenal insufficiency has similar findings but also includes hyperpigmentation from increased CRH and hyperkalemia due to superimposed mineralocorticoid deficiency [1].

Clinicians should not wait for laboratory results of cortisol levels to treat adrenal crisis due to its severity and the grave implications of delaying administration of steroids. Treatment of adrenal crisis involves large doses of intravenous (IV) or intramuscular (IM) doses of hydrocortisone, as well as rapid rehydration with approximately four to six liters of isotonic saline while frequently observing for signs of fluid overload [5]. Recommended doses of steroid therapy include 100 mg of hydrocortisone IV or IM as a bolus dose, followed by continuous IV administration of 200 mg of hydrocortisone over a period of 24 hours [5]. An alternate method includes pulse dosing of 50 mg of hydrocortisone IV or IM every six hours [5]. 

The use of a simulated clinical scenario is a valuable tool in the medical armamentarium whereby learners improve analytical and processing gaps in medical decision-making and diagnostic accuracy [6]. Our case was inspired by an actual patient experience, and the simulation was originally implemented as a neurosarcoidosis scenario. Due to its natural complexity, however, very few residents were able to identify neurosarcoidosis and were stumped as to its diagnosis and treatment. We changed the focus to adrenal insufficiency to create a more effective teaching tool and implemented a new standardized scenario.

It is interesting to note that we incidentally found a simulation module published in MedEd from 2015 in which a patient with sarcoidosis presented with adrenal crisis [7]. This leads us to consider that the presentation of adrenal insufficiency in sarcoidosis may have a higher incidence than originally thought. We have created this module to be an integral part of strengthening and solidifying understanding of adrenal insufficiency in the clinical setting. Our case is unique as we can track residents and medical students’ understanding of adrenal insufficiency with objective tests. With respect to the educational format of the module, our simulation is in an enhanced simulation format with comprehensive and thorough simulation components.

This is a formative activity designed to enhance these cognitive processes, as well as the ability to diagnose and treat adrenal insufficiency. Our case format is useful for first-year internal medicine, family medicine, and emergency medicine residents, as well as upper-level medical students, to improve clinical knowledge and enhance cognitive capabilities.

## Materials and methods

This scenario was conducted during noon conference for four internal medicine residents and two upper-level medical students. Due to the limitations of being in a community hospital, senior internal medicine residents familiar with standardized patient scenarios were recruited to play the roles of the patient and nurse. Prior to the scenario, Dr. Malakooti coached the resident actors and underwent a thorough review of the supplemental materials with them, including the standardized case development tool (Table 1), scenario introduction (Table 2), a note from the husband (Table 3), and a standardized patient scenario (Table 4). Prior to the start of the scenario, a pre-simulation questionnaire was distributed to the residents for self-assessment of their comfort with (and knowledge of) adrenal insufficiency and associated topics (Table 5).

**Table 1:** Standardized Case Development Tool IV: intravenous; EKG: electrocardiogram

Standardized Case Development Tool	
Patient name	Abigail Kingsley
Chief complaint	Weakness and fatigue for one week
Type and level of learner	Residents and upper-level medical students.
Case objectives	Immediately address and treat acute symptoms of hypoglycemia and hypotension
	Identify signs and symptoms of adrenal insufficiency
	Obtain history of presenting illness, medical history, and full pharmacologic history
	Diagnose drug-induced adrenal crisis
	Treat adrenal crisis with appropriate steroids
	Recognize adrenal insufficiency and adrenal crisis along with differential diagnoses
Setting	Emergency Department
Patient profile	
Age	70 years old
Religious background	Baptist
Sex	Female
Sexual orientation	Heterosexual
Patient appearance	Well nourished and in clean casual clothing
Physical limitations	none
Affect	Pleasant but sleepy and at times becomes confused
Social	Lives with her husband of 30 years who is very supportive and takes care of her. He actually knows her medications even better than she does.
Education	Bachelor of Arts degree
Health literacy	Average health literacy
Employment	Previously a stay at home mother
Dwelling	Owns a one story house
Habits	She eats grilled chicken, fish, and vegetables often. She goes on long walks with friends. She does not smoke, drink alcohol, or use illicit substances.
Daily routine	She usually wakes up in the morning and makes eggs and potatoes for her husband and herself, then does some errands until lunchtime. She sees her daughters and their families in the afternoons and evenings and usually has dinner with them.
Case information	
Chief concern	I’m just feeling so tired and weak. I guess I’m just not myself so my husband wanted me to come in and get checked out.
The patient’s story	The weakness and fatigue started about a week ago. It has just been so gradual I don’t remember exactly when it started or what happened. I just feel so sleepy. My husband says I have gotten a little more forgetful this week. We just went on a family vacation to Switzerland where my brother-in-law is working at a huge science facility. I’ve never seen anything like it. It was so beautiful and nice to be with all of our family I just miss everyone so much.
	I ran out of prednisone while we were on our vacation. I haven’t had a chance to refill it yet but I will as soon as I get out of the hospital. My doctor prescribed it for me because I have sarcoidosis.
History of presenting illness	
Onset	Gradually and started one week ago
Setting	When she ran out of prednisone
Duration	Over the past week
Associated with	Sometimes her stomach hurts and she feels nauseous but she hasn’t vomited
Attitude	She believes her symptoms are due to lack of sleep and changing time zones from Switzerland. She thinks if she sleeps more it will get better.
Aggravated by	Moving around and trying to do things, flying back from Switzerland because we had to find all of our suitcases and one was lost.
Relieved by	Laying down and sleeping
Associated with	Sometimes her stomach hurts and she feels nauseous but she hasn’t vomited
Attitude	She believes her symptoms are due to lack of sleep and changing time zones from Switzerland. She thinks if she sleeps more it will get better.
Overall course	Getting worse over the course of the week.
Review of systems: pertinent positive	Dizziness
	Abdominal pain
	Nausea
	Lethargy
	Confusion
Review of systems: pertinent negative	Diarrhea
	Focal weakness
	Loss of consciousness
	Traumatic injury
	Vomiting
Past medical history	Sarcoidosis
Allergies	No known drug allergies
Vaccinations	Up to date with all vaccinations
Surgeries	Two C-sections
Trauma	None
Hospitalization	Hospitalized twice for her two C-sections many years ago
Sexual history	One sexual partner (her husband). They are in a monogamous committed relationship. There is no risk of domestic violence.
Obstetrics and gynecology history	Age of onset of menses: 13
	Age of menopause: 50
	Number of pregnancies: 3
	Number of live births: 2
	Number of miscarriages: 1
	Number of abortions: 0
Medications	Prednisone, 10 mg daily, for sarcoidosis
Immunizations	Tetanus, Influenza, Hepatitis B, Pneumovax
Tobacco products	Never smoker
Alcohol	Never drinker
Illicit drugs	No history of drug use
Diet	Mostly salads, fruits and vegetables, fish and chicken. Eats pork or steak only once or twice a month
Exercise	Does not exercise but does take long walks with her friends from church.
Family history	Father has diabetes
Pertinent physical exam findings	
Layman’s terms	Mild discomfort when the abdomen is pushed down. The pain does not cause the patient to jump or yell out in pain.
General appearance	Well-groomed and dressed in clean casual clothes from home. She is cooperative but gets very sleepy and sometimes confused during history and examination.
Vital signs	Initial vital signs are provided on a sheet at the beginning of the case to the learners:
	Temperature: 98.9 degrees Fahrenheit
	Heart rate: 120 beats per minute
	Blood pressure: 80/62
	Respiratory rate: 14 breaths per minute
	Oxygen saturation: 98% on room air
Specific findings and affect	At times, she forgets what the clinician just asked and says “I’m sorry, what?” or “huh?”.
Diagnosis	Adrenal insufficiency is the most likely diagnosis due to the patient’s history of abrupt discontinuation of steroids with concomitant hypoglycemia and hypotension.
Differential Diagnosis	Hypoglycemia, sepsis
Management	Obtain bedside glucose
	Treat low glucose
	Give IV fluids
	Obtain EKG
	Identify abrupt discontinuation of prednisone
	Start steroids
Challenges	The learners are challenged with obtaining the history of prednisone use that was recently stopped. They must either ask regarding medication history or sarcoidosis management in order for the patient to divulge recent prednisone use that was stopped one week ago.
	The patient is very cooperative but at times forgets what she was saying and/or what questions she was answering. This makes it difficult for the learner to obtain the history. The learners must be persistent in obtaining the full history.
	Once the learners find out about the history of prednisone that was recently stopped, they should immediately start steroid therapy.
	Five minutes into the case, if the learners have not found out regarding prednisone history, the nurse will provide a medication list from the patient’s husband.
	Seven minutes into the case, if the learners have not initiated steroids, the patient will sleep completely through questions.

**Table 2:** Scenario Introduction

Scenario Introduction
Ms. Abigail Kingsley is a 70-year-old female with a past medical history of sarcoidosis.
She has been brought to the emergency department by her husband due to worsening confusion, weakness, dizziness, and fatigue for one week.
Initial vital signs
Temperature: 98.9º Fahrenheit
Heart rate: 120 beats per minute
Blood pressure: 80/62 mm Hg
Respiratory rate: 14 breaths per minute
Oxygen saturation: 98% on room air

**Table 3:** Note From Husband

Note from husband
Abby’s med list:
Prednisone, 10 mg every day (for the past five years)
She forgot to take her pills while we were on our family vacation last week but otherwise she takes it every day. Please help her get better. Thanks doc.
-Richard (Husband) Cell 555-6304

**Table 4:** Standardized Patient Scenario EKG: electrocardiogram; C-sections: caesarean sections

A Sarcoidosis Patient Presents With Adrenal Insufficiency: A Standardized Patient Scenario for Medical Students and Residents		
Patient Name	Abigail Kingsley	
Patient Age	70 years old	
Chief Complaint	Confusion, weakness, dizziness, and fatigue for one week	
Primary Learning Objectives	Immediately address and treat acute symptoms of hypoglycemia and hypotension	
	Identify signs and symptoms of adrenal insufficiency	
	Obtain history of presenting illness, medical history, and full pharmacologic history	
	Diagnose drug-induced adrenal crisis	
	Treat adrenal crisis with appropriate steroids	
	Recognize adrenal insufficiency and adrenal crisis along with differential diagnoses	
Critical Actions	Obtain bedside glucose	
	Treat hypoglycemia intravenously (IV)	
	Address hypotension with IV fluids	
	Obtain EKG	
	Request urine analysis	
	Request blood cultures	
	Identify any changes in medication history	
	Diagnose drug-induced adrenal crisis	
	Initiate pharmacologic therapy with appropriate glucocorticoid therapy	
Learner Preparation		
Initial presentation	A 70-year-old female with a past medical history of sarcoidosis presents with confusion, weakness, dizziness, and fatigue for the past one week. Initially, unbeknownst to the learner, she suddenly stopped taking her chronic prednisone therapy a week prior to presentation. The learner objectives are to identify adrenal crisis and initiate appropriate pharmacologic treatment prior to progression of adrenal crisis.	
Initial vital signs	Temperature: 98.9º Fahrenheit	
	Heart rate: 120 beats per minute	
	Blood pressure: 80/62	
	Respiratory rate: 14 breaths per minute	
	Oxygen saturation: 98% on room air	
Overall Appearance	Patient is lying down in a hospital bed. She is lethargic and falls asleep intermittently. She is in no distress.	
Standardized patient and other roles	At the beginning of the case, an actress plays the role of the patient lying down in the hospital bed. An actor/actress plays the role of the nurse.	
History of Presenting Illness	The learners must elicit the patient’s pharmacologic history of chronic prednisone therapy. The prednisone was suddenly stopped a week prior as she forgot to take her pills with her while traveling for a family vacation.	
Past Medical History	Sarcoidosis	
Past Surgical History	2 C-sections over 30 years ago.	
Home Medications	Prednisone, 10 mg by mouth daily, for five years	
Allergies	No known drug allergies	
Family history	Father had diabetes	
Physical Examination		
General	Laying in the hospital bed. Appears weak and lethargic	
Head, eyes, ears, nose, throat	Normocephalic and atraumatic. Pupils are equally reactive to light and accommodation	
Neck	No abnormal findings	
Lungs	Normal breath sounds	
Cardiovascular	Tachycardia with regular rhythm and no murmur	
Abdomen	Mild abdominal tenderness which is diffuse in all quadrants and there is no rebound or guarding	
Neurological	Awake and oriented and no focal neurologic deficits	
Skin	No lesions	
Genitourinary	No bladder distension	
Psychiatric	Cooperative but falls asleep intermittently during history and examination. At times forgets what the clinician is asking and repeatedly says “Huh?”	
Instructor Notes - change in case and branch points		
Intervention and time point instructions	Change in Case	Additional information
Learners obtain serum glucose	No change	
Learners treat hypoglycemia with IV dextrose	If IV dextrose therapy is not initiated at three minutes, the patient begins to mumble incoherently.	
Learners address hypotension with IV fluids	No change in patient’s mental status. Blood pressure improves to 89/64.	
Learners obtain EKG	EKG image is shown to learners which reveals sinus tachycardia	
Learners obtain urinalysis and request blood cultures	Results are given to learners which show negative results.	
Learners elicit past pharmacologic history revealing chronic prednisone use that was recently stopped due to traveling for a family vacation	No change	
Five minutes after the start of case	If medical history has not yet been obtained, nurse should enter the room	The nurse hands the learners a medication list provided by the patient’s husband
Seven minutes after the start of case	If no steroids are initiated, patient sleeps through questions.	The nurse tells the learners that the patient’s blood pressure is now 70/40 and asks the learners if they would like to start any treatment for the patient.
Learners treat patient with appropriate IV steroid therapy.	Patient begins to talk and states she feels much better and is ready to go home. Case ends.	
Ideal Scenario Flow	Learners enter the hospital room where the patient is lying in bed. They elicit history from the patient which reveals a five-year history of prednisone therapy for sarcoidosis which was abruptly stopped last week as she forgot to take her pills while traveling for a family vacation. The learners treat the patient’s immediate symptoms and obtain appropriate initial tests for a hypotensive and hypoglycemic patient. The learners diagnose adrenal crisis and after treatment, the patient becomes alert, awake, and oriented and states that she feels much better.	
Anticipated Mistakes	Failure to obtain bedside glucose and/or treat with IV dextrose	
	Failure to address hypotension with IV fluids	
	Failure to obtain EKG to exclude cardiac abnormality including myocardial infarction	
	Failure to test for infection with urinalysis and blood cultures	
	Failure to obtain medication history indicating recent medication change: nurse may provide document left behind by the patient's husband	
	Failure to treat with appropriate steroid therapy: nurse may prompt questioning regarding additional medications for treatment	

**Table 5:** Pre-scenario and Post-scenario Assessments MS: medical student; PGY: post-graduate year; TSH: thyroid stimulating hormone; T3: triiodothyronine; T4: thyroxine; ACTH: adrenocorticotropic hormone

PRE-SIMULATION QUESTIONNAIRE						
Instructor:						
Date:						
Which of the following best describes your training level? (please circle)	MS-III	MS-IV	PGY-1	PGY-2	PGY-3	PGY-4 or Fellow
If you are at the postgraduate level, what is your specialty?	Emergency Medicine	Family Medicine	Internal Medicine	Endocrinology	Rheumatology	Other
Have you ever participated in a simulation case before?	Yes	No				
Please rate your own knowledge and comprehension of the following topics:	1 = very poor	2 = poor	3 = neutral	4 = good	5 = very good	
Thyroid storm	1	2	3	4	5	
Diabetic Ketoacidosis	1	2	3	4	5	
Myocardial Infarction	1	2	3	4	5	
Adrenal Insufficiency	1	2	3	4	5	
Pulmonary Embolism	1	2	3	4	5	
Sarcoidosis	1	2	3	4	5	
Which of the following best describes the levels in the following conditions? (please circle)						
	Primary Adrenal Insufficiency	Tertiary (Drug-Induced) Adrenal Insufficiency				
Cortisol	Low / Normal / High	Low / Normal / High				
Cortisol 30 minutes after cosyntropin stimulation test	Low / Normal / High	Low / Normal / High				
Aldosterone	Low / Normal / High	Low / Normal / High				
ACTH	Low / Normal / High	Low / Normal / High				
Which of the following best describes the levels in the following conditions? (please circle)						
	Subclinical Hypothyroidism	Hypothyroidism	Hyperthyroidism			
Serum TSH	Low / Normal / High	Low / Normal / High	Low / Normal / High			
Serum Free T4	Low / Normal / High	Low / Normal / High	Low / Normal / High			
Serum Free T3	Low / Normal / High	Low / Normal / High	Low / Normal / High			
POST-DEBRIEFING / POST- SIMULATION QUESTIONNAIRE						
Please rate your own knowledge and comprehension of the following topics:	1 = very poor	2 = poor	3 = neutral	4 = good	5 = very good	
Adrenal Insufficiency	1	2	3	4	5	
Sarcoidosis	1	2	3	4	5	
Please answer the following questions regarding the patient scenario.						
The simulation case represented a real-life scenario.	strongly disagree	disagree	neutral	agree	strongly agree	
The simulation case was well devised to achieve the goals in the debriefing session.	strongly disagree	disagree	neutral	agree	strongly agree	
The simulation case contributed to or solidified my understanding of important concepts.	strongly disagree	disagree	neutral	agree	strongly agree	
Which of the following best describes the levels in the following conditions? (please circle)						
	Primary Adrenal Insufficiency	Tertiary (Drug-Induced) Adrenal Insufficiency				
Cortisol	Low / Normal / High	Low / Normal / High				
Cortisol 30 minutes after cosyntropin stimulation test	Low / Normal / High	Low / Normal / High				
Aldosterone	Low / Normal / High	Low / Normal / High				
ACTH	Low / Normal / High	Low / Normal / High				
Please comment here if you have any suggestions on how to make this a more effective learning experience:						

The environment was modeled after an emergency room. The patient was found lying down in a hospital bed or stretcher. A blood pressure cuff, heart rate monitor, and oxygen saturation monitor were present, along with an IV pole and simulation bottles of various intravenous medications, including hydrocortisone. A document with an introduction to the scenario was provided to the learners which included the patient’s chief complaint and initial vital signs (Table 2). Lab values were provided on request (Table 6). A supplemental document containing a note from the patient’s husband would be presented to the learners at the fifth minute of the scenario if they had not yet discovered the patient’s past history of long-term prednisone therapy (Table 3). The patient scenario is described in further detail in Table 4. 

**Table 6:** Lab Values CBC: complete blood count; BUN: blood urea nitrogen; TSH: thyroid stimulating hormone; T3: triiodothyronine; T4: thyroxine; pH: potential of hydrogen; RBC/hpf: red blood cells per high-power field; WBC/hpf: white blood cells per high-powered field

Lab values
CBC
Hemoglobin: 13 g/dl
Hematocrit: 46%
White Blood Cell: 6,000/mm^3^
Platelets: 200,000/ mm^3^
Basic Metabolic Panel
Sodium: 125
Bicarbonate: 24
Potassium: 3.1
Chloride: 100
BUN: 45
Creatinine: 1.5 mg/dL
Serum glucose: 60 mg/dL
Point of care serum glucose: 60 mg/dL
Serum cortisol level: 0.5 ug/dl
TSH: 2.0 mIU/L
Free T4: 1.2 ng/dl
Urine drug screen
Opiates: negative
Barbiturates: negative
Methadone: negative
Amphetamines: negative
Cocaine: negative
Marijuana: negative
Urine analysis
pH: 5.0
Color: Clear dark yellow
Specific gravity 1.015
Ketones: none
RBCs: 2 RBC/hpf
WBC: 5 WBC/hpf
Leukocyte esterase: negative
Nitrates: negative
Bacteria: none
Yeast: none
Bilirubin: negative
Blood cultures: No growth

Learners went through the 10-minute standardized patient scenario in groups of two. After the scenario concluded, the instructor discussion guide was used for discussion and debriefing (Table 7). Debriefing involved a case summary from the participants and feedback regarding strengths and areas of improvement for the learners. The critical action checklist (Table 4) was reviewed, and a group discussion was facilitated regarding the clinical presentation and lab abnormalities corresponding with adrenal insufficiency and differential diagnoses. Diagnosis and treatment of adrenal insufficiency were emphasized. After the debriefing session concluded, learners who participated in the case scenario were re-assessed with the post-scenario questionnaire (Table 5).

**Table 7:** Instructor Discussion Guide EKG: electrocardiogram; IM: intramuscular: ACTH: adrenocorticotropic hormone; TSH: thyroid stimulating hormone; T4: thyroxine

Instructor Discussion Guide	
I. Participants	Residents and upper-level medical students
II. Objectives	Immediately address and treat acute symptoms of hypoglycemia and hypotension.
	Identify signs and symptoms of adrenal insufficiency.
	Obtain history of presenting illness, past medical history, and pharmacologic history.
	Diagnose drug-induced adrenal crisis.
	Treat adrenal insufficiency with appropriate steroids.
	Recognize and treat adrenal insufficiency and adrenal crisis along with pertinent differential diagnoses.
III. Role outline	Obtain appropriate history, discuss differential diagnoses and how to appropriately treat the patient with the learners, case summary.
IV. Debriefing	
Learner Evaluation	Discussion with participants regarding their differential diagnoses.
Review critical actions	Obtain bedside glucose.
	Treat hypoglycemia intravenously (IV).
	Address hypotension with IV fluids.
	Obtain EKG.
	Request urine analysis.
	Request blood cultures.
	Identify any changes in medication history and eliciting history of abrupt discontinuation of chronic prednisone therapy.
	Diagnose drug-induced adrenal crisis.
	Initiate treatment with high dose IV or IM steroids.
V. Overview of hypothalamic-pituitary-adrenal axis	Hypothalamus: Secretes Corticotropin-Releasing Hormone (CRH).
	Anterior Pituitary: Releases Adrenocorticotropic Hormone (ACTH).
	Adrenal Cortex: Secretes cortisol.
	Hypothalamus secretes CRH, which stimulates the anterior pituitary to release ACTH, which in turn prompts the adrenal cortex to secrete cortisol.
	Cortisol self-regulates via negative feedback to the hypothalamus and anterior pituitary.
VI. Primary Adrenal Insufficiency	Adrenal gland dysfunction and usually autoimmune (Addison's).
	Loss of both glucocorticoids and mineralocorticoids.
	Hyperpigmentation (due to increased CRH production).
	Hyperkalemia (due to mineralocorticoid deficiency).
VII. Secondary Adrenal Insufficiency	Anterior pituitary dysfunction in regulating cortisol levels.
	Low ACTH levels.
VIII. Tertiary Adrenal Insufficiency	Hypothalamic dysfunction in regulating cortisol levels.
	Low CRH levels.
IX. Drug-Induced Adrenal Insufficiency	Chronic steroid treatment turns off the regulatory mechanism in both the anterior pituitary and the hypothalamus.
	Treatment with prednisone is common in patients with rheumatic diseases.
	Typically occurs in doses > 5 mg of prednisone daily if administered for over one month.
	Chronic use of steroid injections, creams, and inhalers can also cause adrenal insufficiency.
X. Acute Adrenal Insufficiency (Adrenal Crisis) in Secondary and Tertiary Adrenal Insufficiency	Symptoms: Confusion, fatigue, weakness, nausea, vomiting, abdominal pain, dizziness.
	Laboratory Findings: Hyponatremia, Hypoglycemia, Pre-renal failure.
XI. Investigations for Adrenal Crisis	Bedside glucose.
	Blood pressure monitoring.
	EKG to exclude cardiac abnormality including myocardial infarction.
	Test for infection with urinalysis and blood cultures.
	Recent changes in medication history.
	Blood counts and electrolyte counts.
	TSH and free T4.
	Cosyntropin test (Synacthen test or ACTH stimulation test).
	In primary adrenal insufficiency, aldosterone will be low due to additional loss of mineralocorticoids.
XII. Cosyntropin test	Obtain baseline serum cortisol level, then administer ACTH and obtain repeat cortisol level thirty minutes after administration of ACTH.
	Do not need to wait for serum cortisol and ACTH levels to treat if the patient is unstable.
XIII. Differential Diagnoses	Thyroid disorder, Myxedema Coma.
	Hypoglycemia.
	Diabetic Ketoacidosis.
	Gastroenteritis.
	Urinary tract infection.
	Appendicitis.
	Cholelithiasis.
	Sepsis.
	Adrenal insufficiency secondary to sarcoid granulomas.
XIV. Treatment	Treatment of hypoglycemia with IV dextrose.
	Addressing hypotension with four to six liters of isotonic saline while frequently observing for signs of fluid overload.
	Recommended doses of steroid therapy include 100 mg hydrocortisone IV or IM as a bolus dose followed by continuous intravenous administration of 200 mg hydrocortisone over a period of 24 hours.
	An alternate method includes pulse dosing of 50 mg hydrocortisone IV or IM every six hours
XV. Questions to stimulate discussion	What are the differential diagnoses for adrenal insufficiency?
	What did you use to make your final diagnosis of adrenal crisis?
	How would your treatment change if the patient also had an elevated white blood cell count?

Each learner completed the encounter once. On average, the learners took 10 to 15 minutes to complete the pre-scenario questionnaire. The actual standardized patient scenario lasted 10 minutes. The debriefing was completed in under 20 minutes and the post-scenario questionnaire (Table 5) took 10 to 15 minutes to complete. Each case in its entirety concluded in less than one hour.

Learner assessment

We used five-point Likert scales for learner self-assessment, as well as learner evaluation of the module. We constructed an objective written test to determine baseline knowledge of adrenal insufficiency which was compared with a post-scenario test (Table 5). These diverse forms of assessment were used to create a comprehensive understanding of the utility of our module.

## Results

**Figure 1:**
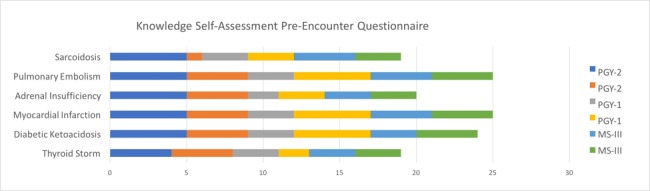
Knowledge Self-Assessment Pre-encounter Questionnaire The X-axis represents cumulative answers from all participants (n = 6) based on a five-point Likert scale for each topic. 1 = Very Poor, 2 = Poor, 3 = Neutral, 4 = Good, 5 = Very Good.

Learners who completed the encounter included two third-year medical students (MS-III), two first-year residents (PGY-I), and two second-year residents (PGY-II). All except for one medical student had completed a simulation case or standardized patient scenario in the past. A five-point Likert scale was used in both pre- and post-assessment questionnaires. In the pre-assessment, when asked regarding their knowledge of six topics, on average, they considered themselves to have the least knowledge of adrenal insufficiency, thyroid storm, and sarcoidosis (Figure 1).

**Figure 2:**
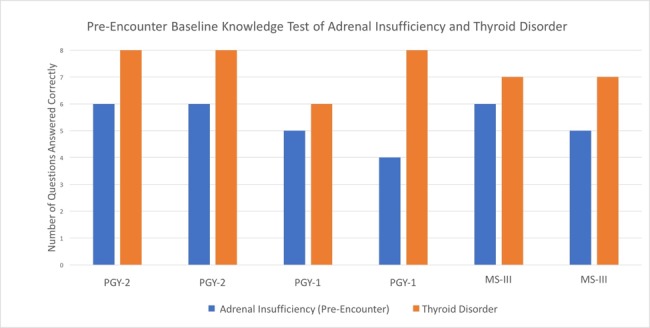
Pre-encounter Knowledge Test Comparison of Adrenal Insufficiency and Thyroid Disorder The X-axis compares adrenal insufficiency and thyroid disorder baseline knowledge tests for each learner. The Y-axis represents the number of questions answered correctly out of eight questions.

To assess objective medical knowledge of adrenal insufficiency and thyroid disorder, the learners completed eight pre-encounter questions for each topic. The average score of the adrenal insufficiency test was 5.33 out of 8 (66.63% correct), and no learner answered more than six out of eight questions correctly. The average score of the thyroid disorder test was 7.33 out of 8 (91.25% correct), and all learners answered at least six out of eight questions correctly (Figure 2).

**Figure 3:**
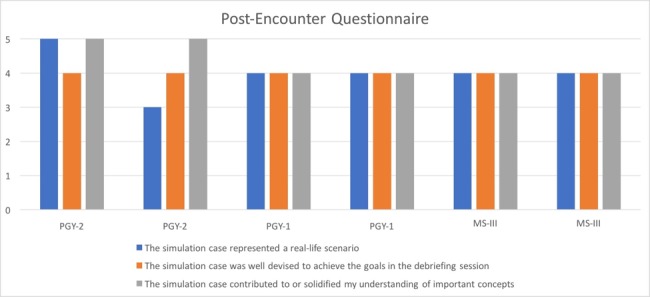
Post-encounter Questionnaire X-axis represents five-point Likert scale. 1 = Strongly Disagree, 2 = Disagree, 3 = Neutral, 4 = Agree, 5 = Strongly agree

In the post-encounter questionnaire, all learners found that the simulation case contributed to their understanding of adrenal insufficiency and that the simulation case was well-devised to achieve this goal (Figure 3). Learner self-assessment of their own knowledge and comprehension of sarcoidosis and adrenal insufficiency improved post-encounter.

**Figure 4:**
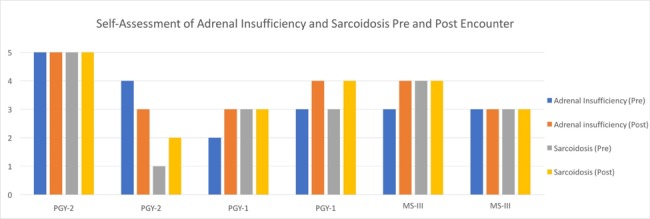
Pre and Post Encounter Self-Assessment Questionnaire. Self-assessment of knowledge and comprehension of adrenal insufficiency and sarcoidosis. The Y-axis represents a five-point Likert scale. 1= Very Poor, 2=Poor, 3= Neutral, 4= Good, 5=Very Good.

When asked regarding their self-assessment of knowledge and comprehension of adrenal insufficiency and sarcoidosis post-encounter and debriefing, overall, the learners’ reported that their knowledge and comprehension had improved in comparison to the pre-assessment (Figure 4).

**Figure 5:**
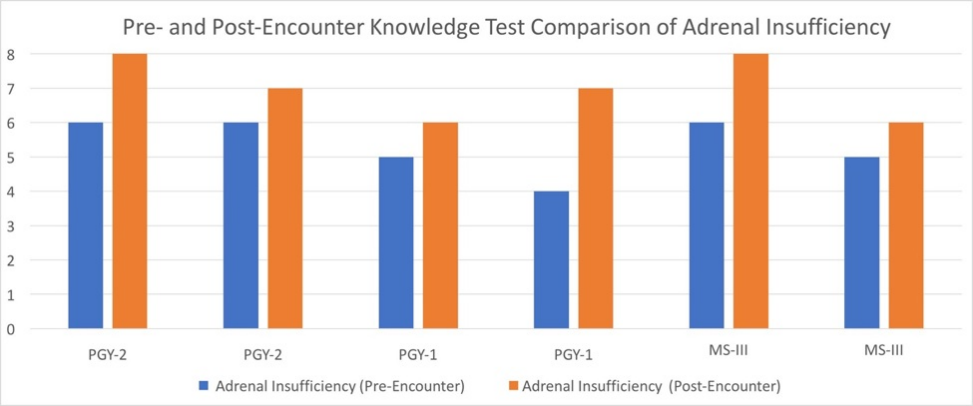
Pre- and Post-encounter Knowledge Test Compares pre- and post-encounter objective knowledge of adrenal insufficiency. The Y-axis represents the number of questions answered correctly out of eight questions possible.

After completing the encounter and debriefing, the learners were asked the same eight pre-encounter questions to re-assess medical knowledge of adrenal insufficiency. The mean score on the adrenal insufficiency knowledge test went up from an average of 66.63% to 87.50% correct (Figure 5).

## Discussion

In the pre-assessment, we asked the learners whether they had more knowledge of thyroid disease or adrenal insufficiency, and they were more confident in their knowledge of adrenal insufficiency. It was interesting to note that there was a discrepancy between the self-assessment and objective assessment. In the pre-assessment, learners overall answered 91.25% of questions correctly regarding thyroid disorder, but only 66.63% of adrenal insufficiency questions were answered correctly. The learners were initially overconfident in adrenal insufficiency and less than confident in thyroid disorder despite objective tests of medical knowledge indicating that they were less knowledgeable in adrenal insufficiency and had good knowledge of thyroid disorder.

During the debriefing session, the learners indicated that they were surprised that they were not able to diagnose adrenal insufficiency quickly from the onset of the simulation. We discussed the classification of primary, secondary, tertiary, and acute adrenal insufficiency, and how to differentiate them clinically. Defining types of adrenal insufficiency unexpectedly became the main focus due to extensive questions from the participants. There was less discussion regarding treatment of adrenal insufficiency. During our debriefing, the learners indicated they were more comfortable with initiating treatment with steroid therapy than they were in making the diagnosis.

Overall, the first-year internal medicine residents had the least knowledge of adrenal insufficiency and improved the most on our objective tests. This unexpected finding may be due to the third-year medical students having recently completed a knowledge-based medical board examination. Another possible explanation is the flexibility of fourth-year medical school resulting in variations in baseline knowledge in the months prior to starting residency training. As a result of these findings, this module may be best suited for first-year residents in internal medicine, emergency medicine, or family medicine, and upper-level medical students. Alternatively, it could be used for upper-level residents who may require remediation. Our case can be utilized alongside a MedEd adrenal insufficiency workshop to solidify basic science concepts [8].  

A challenge to this approach is in representing a real-life scenario during the enactment of the scene. In hospitals that do not have standardized patients or simulation centers, it is important to fully prepare and guide the individual who will be performing as the patient in the scenario. For the sake of realism, the individual should develop a familiarity with the script, as well as rehearse the scenario prior to enacting the simulation with the learners. As noted previously, we incidentally found a similar simulation scenario published in MedEd from 2015 in which a patient with sarcoidosis presents with adrenal crisis [7]. It is reasonable to consider that sarcoidosis predisposes patients to adrenal insufficiency in ways that we had not originally anticipated. Further investigations of hospitalized patients with adrenal insufficiency may determine whether sarcoidosis is correlated with an increased incidence of adrenal insufficiency than in other disease processes when chronic steroids are abruptly discontinued.

## Conclusions

Overall, feedback from our residents as determined in a five-point Likert scale, as well as objective tests of knowledge, showed we were able to improve knowledge and comprehension of adrenal insufficiency within a short period of time using a learner-based scenario. The success of our simulation in developing participants’ medical knowledge adds to current evidence showing that using simulation scenarios as an adjunct to teaching improves education and enhances comprehension.
